# Developmental and Intelligence Quotient in Autism: A Brief Report on the Possible Long-Term Relation

**DOI:** 10.3390/bs12090304

**Published:** 2022-08-25

**Authors:** Assia Riccioni, Martina Siracusano, Lucrezia Arturi, Claudia Marcovecchio, Valentina Postorino, Leonardo Emberti Gialloreti, Luigi Mazzone

**Affiliations:** 1 Systems Medicine Department, University of Rome Tor Vergata, 00133 Rome, Italy; 2 Child Neurology and Psychiatry Unit, Department of Neurosciences, Policlinico Tor Vergata Foundation Hospital, 00133 Rome, Italy; 3 Department of Biomedicine and Prevention, University of Rome Tor Vergata, 00133 Rome, Italy; 4 JFK Partners, Department of Pediatrics, School of Medicine, Anschutz Medical Campus, University of Colorado, Aurora, CO 80045, USA

**Keywords:** autism spectrum disorder, cognitive, intelligence, development, assessment, evaluation

## Abstract

Developmental level and cognitive skills assessment represents a crucial aspect in the delineation of the clinical phenotype and long-term outcomes of individuals with autism spectrum disorder (ASD). Nevertheless, the evaluation of cognitive development trajectory across a lifespan ranging from birth to school age appears challenging for clinicians and researchers, because of the lack of measures that coherently cover this timeframe. Thus, the main goal of this community-based study was to investigate within a sample of ASD children if the developmental quotient (DQ), evaluated through the Griffiths Mental Development Scales Extended Revised (GMDS-ER) scale, predicts the non-verbal brief intelligence quotient (IQ), measured through the Leiter-R at follow-up. The main observation of our study was a positive correlation between the level of DQ and nonverbal IQ at follow-up evaluations, highlighting that ASD children characterized by a greater developmental profile will later present higher non-verbal IQ.

## 1. Introduction

Autism spectrum disorder (ASD) is an early-onset neurodevelopmental condition characterized by persistent social and communication impairment in addition to restricted and stereotyped patterns of interests and behaviors [1]. Developmental level and cognitive functioning have proven to be crucial predictors of outcome in ASD [2,3]. It is well known that lower intelligence quotient (IQ) is associated with increased behavioral difficulties and risk of psychiatric comorbidities in this population, leading to greater impairments, strongly impacting a family’s quality of life [4,5,6,7].

In this context, the assessment of the developmental profile and cognitive skills in ASD children assumes a key role in clinical practice, particularly at early stages. Usually, the administration of developmental standardized play-oriented scales (e.g., Griffiths, Bayley), which provide a global developmental quotient (DQ), represents the first choice for the evaluation of preschoolers and/or non-cooperative children with ASD. Whereas, for the IQ assessments—generally performed for school-aged children with a good level of cooperation—the use of a non-verbal cognitive scale, such as the Leiter International Performance Scale-Revised (Leiter-R) [8], is preferred due to the wider possibility of employment within ASD populations (frequently characterized by limited or lacking verbal skills).

The developmental profile has been identified as a positive predictor of cognitive skills within typical development individuals and vulnerable pediatric samples (follow-up of preterm and low-weight newborns, deaf children before and after cochlear implantations) [9,10,11,12,13,14,15]. Whereas the prognostic value of developmental measures for later IQ has not been widely investigated in relation to ASD [16]. Indeed, most of the available data showed a positive concordance between DQ and IQ when performed in the context of the same evaluation, without investigating the long-term relation between these measures [17,18,19,20].

The present study aims to investigate within a clinical setting if developmental quotient– evaluated through the Griffiths Mental Development Scales-Extended Revised (GMDS-ER) [21]—predict the non-verbal brief intelligence quotient measured by the Leiter-R-at follow-up evaluations in a sample of ASD children.

## 2. Methods

This is a community-based study performed in the context of the clinical activity of the Child Psychiatry Unit of the University of Rome Tor Vergata Hospital, which usually provides a developmental assessment (DQ) at first evaluation and cognitive assessment (IQ) at follow-up examinations. Children with ASD referring to our unit undergo clinical evaluations including a measure of ASD symptoms (ADOS-2) and a developmental or cognitive assessment depending on age, language skills and level of participation (i.e., attention, behavioral problems). A DQ evaluation—through the Griffiths Mental Development Scales-Extended Revised (GMDS-ER) [21]—is commonly performed for preschoolers and non-cooperative children, whereas we generally perform a cognitive evaluation for school-aged children with a good level of cooperation.

The present study was conducted according to the guidelines of the Declaration of Helsinki (Ethical Committee approval: #77/13; #146/16).

### 2.1. Participants

The convenience sample of our study was constituted by children diagnosed with ASD, recruited among those followed by our clinical unit. The assessment measures provided for the study were performed in the Child Psychiatry Unit of the Tor Vergata Hospital in the context of routine clinical evaluations. To be included in the study participants were required to have: a diagnosis of ASD according to the Diagnostic and Statistical Manual of Mental Disorders—fifth edition (DSM-5) [1], supported by the Autism Diagnostic Observation Schedule—second edition (ADOS–2) [22]; a developmental evaluation performed with the GMDS-ER [21]; an assessment at follow-up of non-verbal brief IQ measured with the Leiter-R scale [8]. Children with incomplete developmental/cognitive evaluation were excluded. For the purpose of this study, we defined as T0 the day on which the DQ was assessed (GMDS-ER) and, as T1 the day of the subsequent IQ assessment (Leiter-R).

### 2.2. Statistical Analyses

Independent sample t-tests were performed to evaluate sex differences in DQ at the baseline. Spearman’s correlations were used to evaluate relations between quantitative variables. Partial correlations, introducing as control variables the T0-T1 time difference and/or age, were performed to analyze the relationships between DQ and IQ. To further explore associations, while controlling for other variables, a multivariable regression model was used. In a linear regression, with the total IQ score as a dependent variable, DQ, T0-T1 time difference, and age, were entered as independent variables. An alpha level of 0.05 was used for all statistical analyses. Results are reported as means ± Standard Deviations (SDs) if not otherwise specified. All analyses were performed using the Statistical Package for Social Sciences SPSS software (version 26, Inc., Chicago, IL, USA) [23].

### 2.3. Results

A final sample of 143 ASD individuals was included in the study (114 males, 29 females, age at T0 4.4 ± 1.3 years). Mean time difference between T0 (developmental evaluation: GMDS-ER) and T1 (non-verbal IQ evaluation: Leiter-R) was 2.2 ± 1.0 years (Median: 2.2; Interquartile Range: 1.3–3.0). At T0 the median ADOS-Calibrated Severity Score (CSS) was 5 (moderate level of ASD severity) and the median GMDS/ER-DQ total score was 75 (mild development delay). Mean non-verbal brief IQ at T1 was 90.4 ± 23.7. A mean score difference of 14.8 ± 17.2 was observed between IQ at T1 and DQ at T0. No statistically significant differences were found in DQ scores or T0-T1 time differences between genders.

We observed a high positive correlation between GMDS/ER DQ at T0 and Leiter-R IQ at T1 (Spearman’s r = 0.728; *p* < 0.001). Partial correlations as well, while controlling for T0-T1 time distance or both time distance and age, yielded statistically significant correlations between GMDS/ER DQ at T0 and Leiter-R IQ at T1 (r = 0.694, *p* < 0.001 and r = 0.607, *p* < 0.001 respectively) (Table 1).

A multivariable linear regression was calculated to evaluate the relation between DQ and IQ, while adjusting for T0-T1 time difference and age. A statistically significant regression equation was found (F (3139) = 73.788, *p* < 0.001), with an adjusted R^2^ of 0.606. All the three variables were statistically significant predictors of the Leiter-R IQ at T1, with a standardized β of 0.545 (*p* < 0.001) for the DQ score, β = −0.117 (*p* = 0.038) for T0-T1 time difference, and β = −0.353 (*p* < 0.001) for age (Table 1).

## 3. Discussion

Our study showed a positive correlation between the level of DQ and nonverbal IQ at follow-up, meaning that ASD children characterized by a greater developmental profile present later higher non-verbal IQ. This is concordant with other studies, which reported correlations between developmental profiles and cognitive skills in ASD [17,18]. However, several methodological issues do not allow accurate comparisons. Primarily because other authors did not employ the same instruments as ours to measure children’s developmental profiles (Psychoeducational Profile, Third Edition-PEP-3, Kyoto Developmental scale) and IQ (Wechsler Preschool and Primary Scale of Intelligence-WPPSI). Additionally, the developmental and cognitive scales were performed during the same evaluation and not, as in our study, at different time-points. Thus, these studies provide a more cross-sectional clinical picture rather than contributing in outlining a developmental and cognitive trajectory of children with ASD.

Overall, our findings suggest that the assessment of DQ using the GMDS-ER at early stages of development could support the clinician in forecasting a later cognitive outcome. This topic assumes a critical role in daily clinical practice—not only in terms of targeted intervention but also in terms of impact on the quality of life of families. It is well known that parents of children with ASD and low IQ show greater levels of stress [7]; thus, our study may contribute to providing a more evidence-based answer to the recurrent questions parents ask about future intelligence and cognitive trajectories.

We also observed that individuals with ASD included in our sample presented at follow-up a higher IQ, if compared to the DQ (mean 75) measured on average two years earlier. This discrepancy between scores may be explained by the fact that the Leiter-R, as opposed to GMDS-ER, excludes the assessment of language skills—often compromised in individuals with ASD [2]. This may lead to a worse performance in the developmental test.

Our study presents with some strengths. The sample size is larger in comparison to previous studies [17,18] with a preserved male to female ratio (4:1) and the inclusion of both verbal and nonverbal individuals. However, several limitations should be considered when interpreting our data. The time-range distance between T0 and T1 was wide, even if without a major influence on our results. In addition, we only measured the Brief non-verbal IQ of the Leiter-R (instead of the Leiter-R full non-verbal IQ or the full verbal IQ provided by other cognitive scales) due to the limited level of cooperation and language skills of the included children. Finally, an evaluation of adaptive skills was not provided and the possible role of other factors influencing the outcome (such as type/duration/frequency of intervention) was not taken into consideration.

Overall, our study offers evidence of the possible use of GMDS-ER DQ as a predictor of non-verbal cognitive outcome; however, further studies are needed to explore whether the possible role of DQ as a predictor of IQ can be applied even to verbal cognitive skills, also including adaptive behavior.

## 4. Conclusions

Early assessment of developmental and cognitive skills is a critical topic in the definition of clinical phenotype and later outcome in children with ASD. Our results point to DQ—measured through the GMDS-ER− as a useful predictor of non-verbal cognitive outcomes in ASD individuals.

Thus, future research with ASD individuals, including a long-term evaluation of adaptive skills and full IQ assessment, is needed, in order to better explore the usefulness of DQ in predicting later IQ.

## Figures and Tables

**Table 1:** Correlation between baseline DQ (T0) and subsequent nonverbal IQ (T1).

	IQ			
	*Spearman’s correlation*		*Multivariable (Adjusted R^2^ = 0.606)*	
	*r value*	*p-value*	*β*	*p-value*
***DQ***	0.728	<0.001	0.545	<0.001
***T0-T1 time difference***	0.694	<0.001	0.117	0.038
***Age***	0.607	<0.001	0.353	<0.001

**Legend:** DQ: Developmental Quotient; IQ: Intelligence Quotient.

## Data Availability

Data supporting the findings of this study can be available on request from the corresponding author (MS).
